# MediPlEx - a tool to combine in silico & experimental gene expression profiles of the model legume Medicago truncatula

**DOI:** 10.1186/1756-0500-3-262

**Published:** 2010-10-19

**Authors:** Kolja Henckel, Helge Küster, Leonhard J Stutz, Alexander Goesmann

**Affiliations:** 1 Bioinformatics of Signaling Networks, Center for Biotechnology, Bielefeld University, Germany; 2 Unit IV - Plant Genomics, Institute for Plant Genetics, Leibniz Universität Hannover, Germany; 3 Computational Genomics, Center for Biotechnology, Bielefeld University, Germany; 4 Technical Faculty, Bielefeld University, Germany

## Abstract

**Background:**

Expressed Sequence Tags (ESTs) are in general used to gain a first insight into gene activities from a species of interest. Subsequently, and typically based on a combination of EST and genome sequences, microarray-based expression analyses are performed for a variety of conditions. In some cases, a multitude of EST and microarray experiments are conducted for one species, covering different tissues, cell states, and cell types. Under these circumstances, the challenge arises to combine results derived from the different expression profiling strategies, with the goal to uncover novel information on the basis of the integrated datasets.

**Findings:**

Using our new analysis tool, MediPlEx (MEDIcago truncatula multiPLe EXpression analysis), expression data from EST experiments, oligonucleotide microarrays and Affymetrix GeneChips^® ^can be combined and analyzed, leading to a novel approach to integrated transcriptome analysis. We have validated our tool via the identification of a set of well-characterized AM-specific and AM-induced marker genes, identified by MediPlEx on the basis of *in silico *and experimental gene expression profiles from roots colonized with AM fungi.

**Conclusions:**

MediPlEx offers an integrated analysis pipeline for different sets of expression data generated for the model legume *Medicago truncatula*. As expected, *in silico *and experimental gene expression data that cover the same biological condition correlate well. The collection of differentially expressed genes identified via MediPlEx provides a starting point for functional studies in plant mutants. MediPlEx can freely be used at http://www.cebitec.uni-bielefeld.de/mediplex.

## Background

*Medicago truncatula *is a model plant for the functional analysis of legume biology [1]. The ability to interact with beneficial microbial organisms leading to the formation of nitrogen- fixing root nodules [2] and to phosphate-acquiring arbuscular mycorrhizal (AM) roots [3] is one of the main distinctive features of the legume family. AM interactions between the host root and the fungal partner are a particularly interesting field of research, since more than 80% of land plants depend on an efficient AM for the uptake of nutrients, primarily phosphate [4]. By recruiting the basic genetic programme allowing microbial infection during AM [5], legumes such as *Medicago truncatula *evolved the capacity to enter a second beneficial interaction: the nitrogen-fixing symbiosis with the soil bacterium *Sinorhizobium meliloti *[6]. Symbiotic nitrogen fixation allows legume plants such as *Medicago truncatula *to grow on nitrogen-depleted soils and to develop protein-rich seeds, properties exploited in sustainable agriculture. Likewise, apart from direct advantageous effects resulting from an improved plant nutrition, an important indirect benefit of mycorrhization is an enhanced resistance against different abiotic and biotic stress conditions [7].

The great interest in transcriptome studies in *Medicago truncatula *(more than 500 publications in Pubmed [8] by searching for "*Medicago truncatula*" as keywords and the last 5 years as publication time span) is evidenced by the generation and sequencing of more than 70 cDNA libraries, in total yielding more than 250.000 ESTs stored in the DFCI Medicago Gene Index [9]. Parallel to the generation of EST data, thousands of oligonucleotide microarrays were hybridized with targets from different biological conditions [10], using layouts such as Mt16kOLI1 [11] and Mt16kOLI1Plus [12] (Arrayexpress ID: A-MEXP-85/A-MEXP-138). In the last couple of years, Affymetrix Medicago GeneChips^® ^more and more moved into the focus of Medicago transcriptomics, since these more genome-wide tools allow a better comparison of gene expression data from a multitude of conditions [13], leading to more accurate results. Parallel to the development and use of transcriptomics tools, a genome project was conduced for *Medicago truncatula *[14,15].

Different institutes store the various sequence and expression datasets, using them for further analysis, or offering them as downloads. At the J. Craig Venter Institute (TIGR before 2006) EST libraries are clustered and assembled, resulting in species-specific Gene Indices [9] for over 100 species. These GeneIndices, including the *Medicago truncatula *GeneIndex 10.0, are now hosted at the Dana-Farber Cancer Institute (DFCI) [16]. Storing information on how the ESTs were assembled, the GeneIndices allow to relate EST data to the biological conditions used for the generation of cDNA libraries, whilst statistical methods were developed to assess if a gene is differentially expressed under a given condition [17,18]. In contrast to EST data, a range of different databases such as GEO [19,20], Arrayexpress [21], PEPR [22], The Stanford MicroArray Database [23], and PlexDB [24] store microarray and GeneChip^® ^expression data, offering researchers public access to results from transcriptomics experiments. In case of *Medicago truncatula*, the Medicago Gene Expression Atlas [13] has developed into a popular resource for expression profiles relying on Medicago GeneChips^®^.

To yield novel insights into gene expression, it would be desirable to integrate different kinds of *in silico *and experimental expression data. In case of the model legume *Medicago truncatula*, the TRUNCATULIX data warehouse [25] currently integrates five different sequence databases (MtGI 8.0 [9], MtGI 9.0 [9], *Medicago truncatula *454 sequencing project [26], *Medicago truncatula *genome project 2.0 [27], Medicago GeneChip^® ^reporter sequences) as well as oligonucleotide microarray and GeneChip^® ^expression experiments from different source databases. The user can quickly scan the complete database for the expression of genes of interest, but downstream analyses of expression data cannot be performed inside the warehouse. This lack of an integrated expression analysis with an easy-to-use interface and a database connection prompted us to create MediPlEx (MEDIcago truncatula multiPLe EXpression analysis). We here report on the design and implementation of this tool and provide a first example for its use to identify genes activated in *Medicago truncatula *AM roots.

## Results and Discussion

### Software solution

To combine the different kinds of gene expression datasets, we created an analysis tool called MediPlEx. It can be launched via SAMS [28], a Sequence Analysis and Management System, that stores data on Tentative Consensus sequences (TCs = assembled ESTs). Loading the SAMS project for *Medicago truncatula*, users can start a combined expression analysis. To do so, the user first selects the cDNA libraries covering interesting biological conditions. Subsequently, MediPlEx gathers information on the composition of the relevant TCs from SAMS and calculates logarithmic likelihood ratios [17] (c.f. Methods Section), an *in silico *expression measure, for the selected TCs. The microarray experiments to be related to the EST expression data are selected during the next step. Afterwards, MediPlEx fetches the different expression datasets from the TRUNCATULIX data warehouse [25] that stores a variety of expression data being publicly available for *Medicago truncatula*. The results are clustered hierarchically and can subsequently be browsed in an interactive 3D visualization tool implemented in Java [29]. An export option offers the possibility to store the results of the combined expression analysis. A complete list of expression values can be viewed and the result of the hierarchical clustering is shown in a dendrogram. The combined search for gene expression on the basis of EST frequencies and microarray/GeneChip^® ^hybridization data offers the possibility to exploit both *in silico *and experimental expression profiles of various sources to trace novel candidate genes for the biological condition of interest.

### Biological findings

**Figure 1:**
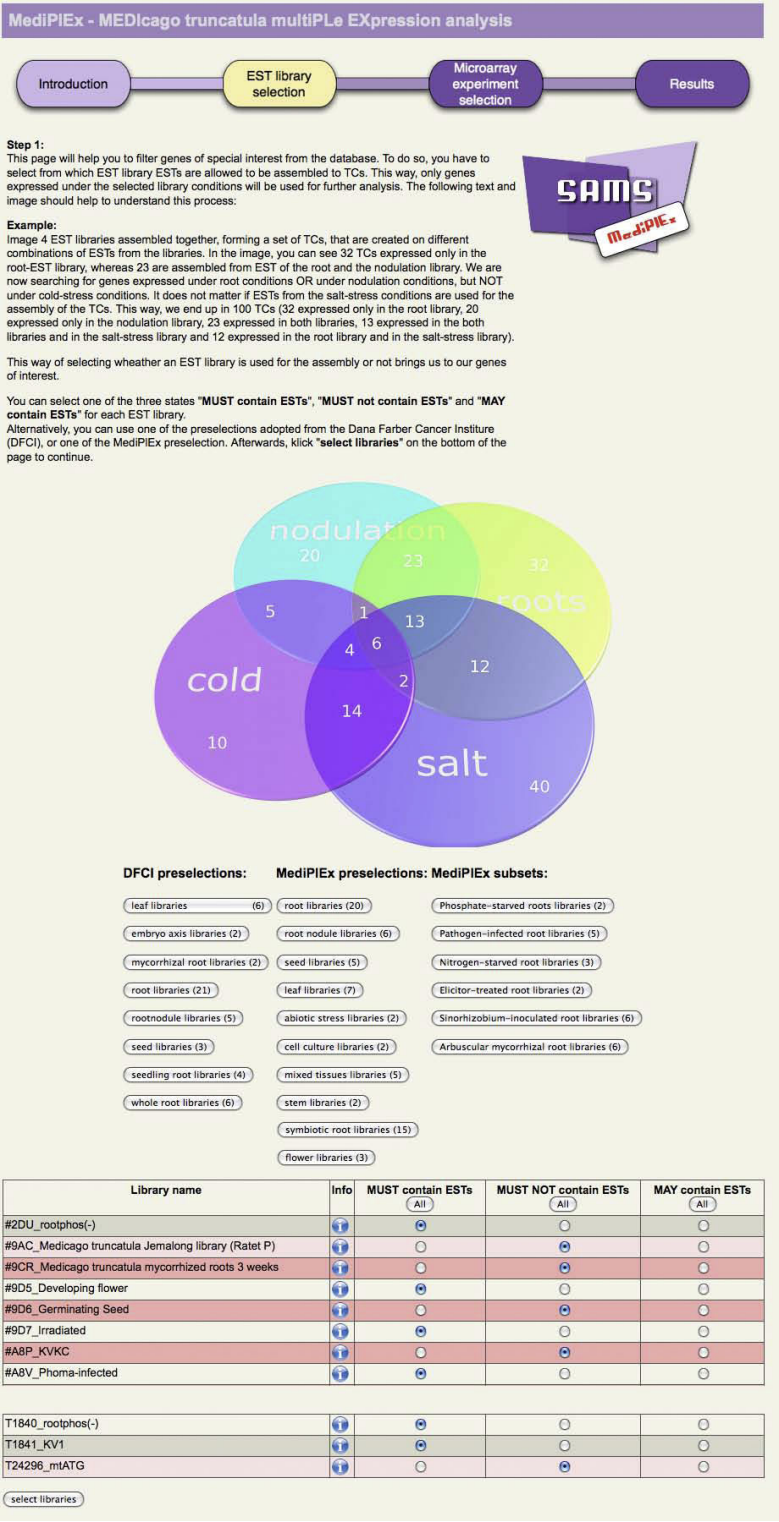
**EST library selection**. The screenshot shows the MediPlEx web page to select the genes of interest by using the EST library assembly. For each library, the user can select one of the three states: 'MUST contain ESTs', 'MUST NOT contain ESTs', and 'MAY contain ESTs'. 'MUST contain ESTs' means that the resulting TCs have to consist of at least one EST from these libraries. 'MUST NOT contain ESTs' denotes that the resulting TCs are not allowed to have an EST assembled from these libraries at all, and 'MAY contain ESTs' indicates that it does not matter if an EST is assembled from these libraries to a TC or not (see venn diagram). EST library preselection are implemented via buttons, the complete library list is available on the bottom of the page. The EST library list is shortened for this screenshot.

We compared the expression based on a selection of different EST-libraries to GeneChip^® ^and microarray analyses performed in the same biological background, in this case the AM symbiosis (Figure 1). To identify AM-specific TCs (and thus AM-specific genes), we used the preselection "arbuscular mycorrhizal root libraries (6) ", consisting of the following selection of EST libraries from the DFCI Medicago GeneIndex:

• MUST contain ESTs (using 'OR' as concatenation):

• #9CR (Medicago truncatula mycorrhized roots 3 weeks)

• #ARB (MTGIM)

• #ARE (MTAMP)

• #GFS (MHAM2)

• 5520 (MtBC)

• T1682 (MHAM)

MAY contain ESTs (IGNORE):

• #IP8 (NOLLY)

• T10174 (kiloclone)

• T11958 (MTUS)

• T12308 (6KUG)

**Figure 2:**
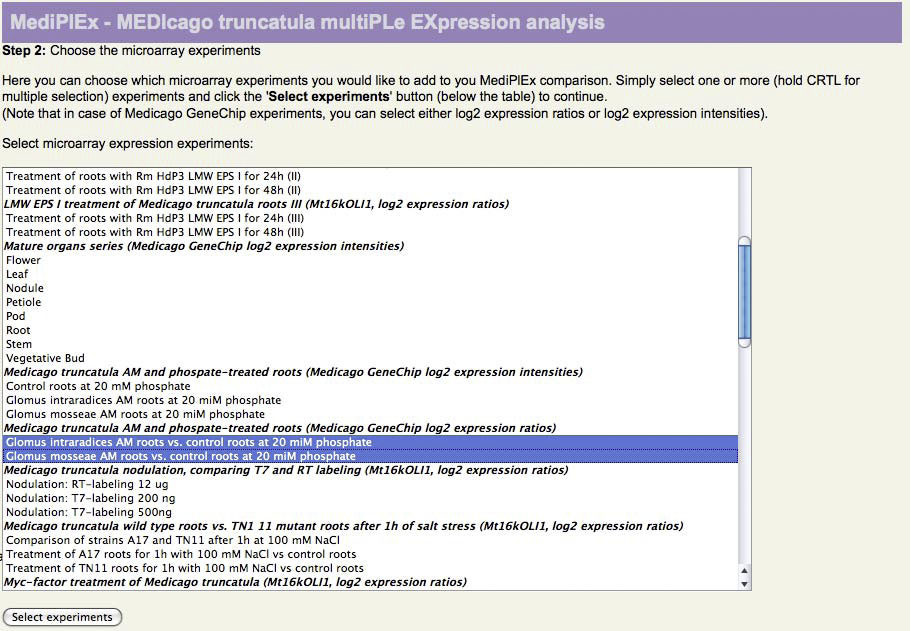
**Microarray selection**. The screenshot shows the selection of microarray experiments (oligonucleotide and GeneChip^®^) to be combined to the EST expression analysis. The user can select as many experiments as desired.

**Figure 3:**
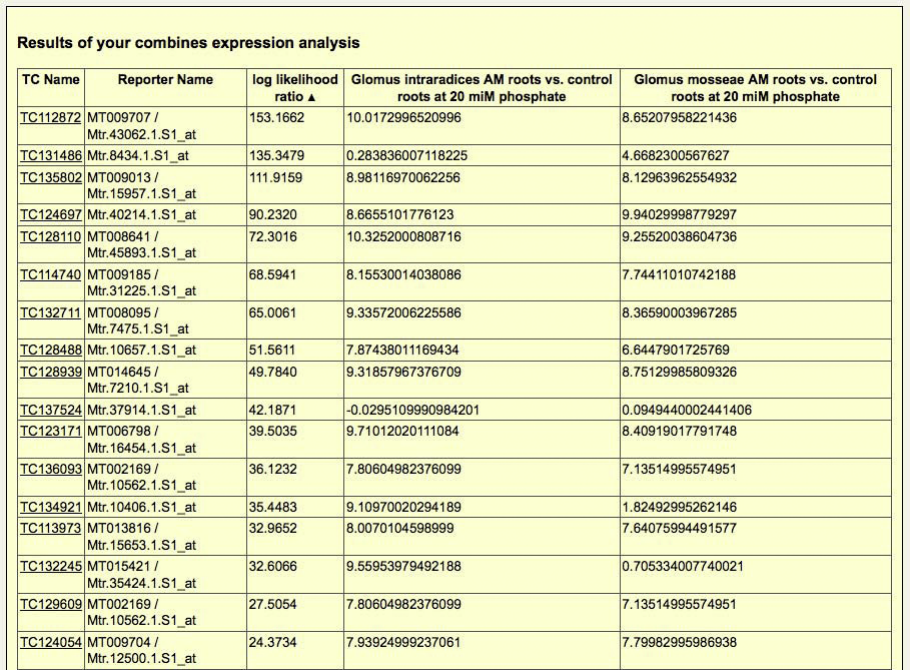
**Resulttable**. The screenshot shows the table listing the *in silico *calculated logarithmic likelihood ratio, as well as the expression datasets of the microarray experiments.

The libraries set to "MAY contain ESTs" were not considered since these mostly represent clone libraries used for microarray construction and thus do not contain information on tissue-specific gene expression. The Medicago GeneChip^® ^datasets selected are derived from the experiment "*Medicago truncatula *AM and phospate-treated roots (Medicago GeneChip log2 expression ratios)", specifically the "*Glomus intraradices *AM roots vs. control roots at 20 miM phosphate" and "*Glomus mosseae *AM roots vs. control roots at 20 miM phosphate" datasets (shown in Figure 2). Following the TC search, 763 TCs fulfilled the specified conditions of an AM-specific EST composition [Additional file 1], and 751 of these were represented by reporters on the Affymetrix Medicago GeneChip^® ^(see Figure 3). Sorting these TCs for the calculated logarithmic likelihood ratio R [17] that provides a measure for differential gene expression under the given conditions, we identified a range of AM marker genes [10,11,30], as was suggested by our search. Remarkably, a TC encoding the mycorrhiza-specific phosphate transporter MtPt4 (TC142142), a key marker gene for an efficient AM symbiosis [31], was identified as the top candidate. In addition, the identification of well-known AM-specific and AM-induced marker genes such as MtBcp1 (TC170722 [11]), MtGlp1 (TC153539 [32]), MtGst1 (TC166174 [33]), MtLec5 TC143161 [34]), MtMYBCC (TC146022 [10]), MtScp1 (TC143816 [35]), MtTi1 (TC152603 [36]) can be regarded as a proof-of-principle for the MediPlEx search strategy.

**Figure 4:**
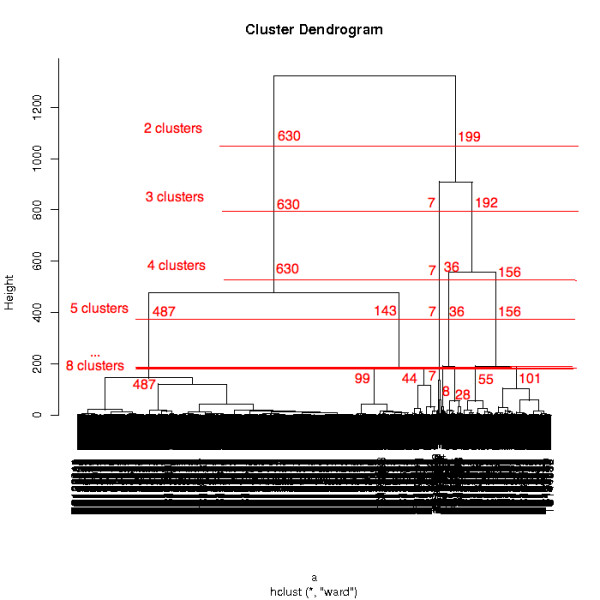
**Cluster dendrogram**. The cluster dendrogram created by the hierarchical clustering (ward clustering) of the expression profiles. The lines indicate which clusters are created when using the different cluster options. The sizes of the clusters are denoted at the specific branches.

**Figure 5:**
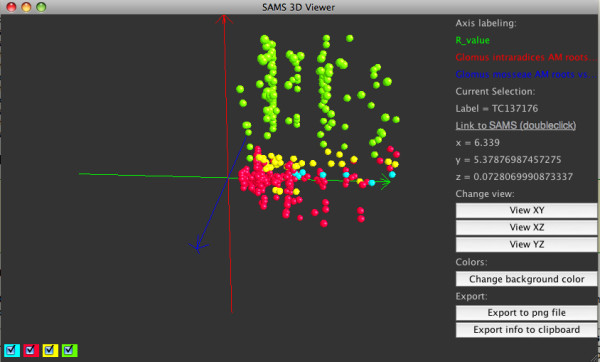
**3D Visualization**. A screenshot of the interactive 3D application created for the visualization of the MediPlEx results. Genes are painted as spheres in the coordinate system, different colors represent the associated cluster. Clicking on one of the spheres, additional information about the gene (label, expression values, html-link to SAMS) is shown. Each cluster can be activated and deactivated for hiding the corresponding genes in order to gain a better overview. The coordinate system is rotatable and zoomable, a snapshot of the 3D view can be saved by clicking the "Save as png file"-button.

In general, the different expression values obtained by *in silico *and experimental expression analysis correlated very well. According to the dendrogram of the hierarchical clustering (Figure 4), we subsequently identified four clusters of expression profiles (the created clusters can be found in [Additional file 2]). Alternatively, clusterings with 2-8 cluster were generated, and the sizes of these can be found in Table 1. The generation of the cluster is demonstrated in the dendrogram in Figure 4. The red lines indicate the positions where the clustertree is cut, the size of the resulting cluster is denoted at these positions. A 3D visualization can be started after selecting the three experiments for the three axis. Figure 5 shows the 3D visualization and the four cluster obtained in different colors.

**Table 1:** The sizes of the different clusters on the performed clusterings.

cluster	cluster1	cluster2	cluster3	cluster4	cluster5	cluster6	cluster7	cluster8
2 cluster	530	221						
3 cluster	530	13	208					
4 cluster	530	13	79	129				
5 cluster	109	421	13	79	129			
6 cluster	109	421	2	11	79	129		
7 cluster	109	421	2	11	79	42	87	
8 cluster	109	421	2	11	21	58	42	87

**Total TCs**	**751**							

The table shows the number of TCs according to the number of clusters created in the expression experiment.

Clustering of expression profiles reveals the predominant activation of different GO categories, e.g. ATP binding (5524), protein amino acid phosphorylation (6468), metabolic process (8152), binding (3677) and nucleus (5634) for cluster 1, transport (6810), DNA binding (3677), integral to membrane (16021), ATP binding (5524) and transporter activity (5215) for cluster 3, and intracellular (5622), structural constituent of ribosome (3735), translation (6412), and ribosome (5840) for cluster 4. These functional differentiations could indicate the fine-tuning or coregulation of specific cellular functions during fungal colonization of AM roots. A list of GO categories for the clustered genes can be found in [Additional file 3].

## Discussion

Many EST and microarray experiments have been performed throughout the last years, leading to an immense amount of expression datasets. Our newly developed application, MediPlEx, integrates two different gene expression analysis methods (EST- and microarrays/GeneChip^® ^-based transcriptome profiling), and delivers new results that have the potential to yield novel insights into gene expression in the model legume *Medicago truncatula*. Similar to MediPlEx, expression analyses can be performed using Simcluster, a tool developed by Vencio in 2007 [37]. Simcluster can take different expression experiment datasets, which include SAGE [38], MPSS [39], and Digital Northern powered by traditional [40] or, recently developed, EST sequencing-by-synthesis (SBS) technologies [41], and map them to the simplex space [42,43]. This mapping should make the data from different data sources and methods more comparable, as the simplex space does not use absolute values and scales, but relative (relative values to the overall expression for single experiments). Unfortunately, Simcluster is not connected to any database, so candidate genes and expression values have to be searched for elsewhere and converted to fit the designated format.

Thus, the database connection for obtaining expression data, combined with an easy-to-use web interface, is a major benefit of the MediPlEx tool. MediPlEx is currently available for two *Medicago truncatula *SAMS projects (SAMS_Medicago_truncatula_DFCI_9 & SAMS_Medicago_truncatula_DFCI_10). Datasets of upcoming expression analysis methods, such as SAGE or MPSS could be integrated as well, that way taking into account also the results of recently developed high-throughput expression profiling strategies.

## Conclusions

The newly developed analysis tool MediPlEx offers an approach for combining gene expression values from already performed expression experiments in order to find candidate genes. By relating different experiments, the user can analyze and cluster transcriptomics data, visualize gene expression in 3D and find cluster of genes with correlating expression. Using our method, existing experimental results can be validated and novel insights into the expression of *Medicago truncatula *genes can be found. The collection of differentially expressed genes identified via MediPlEx provides a starting point for functional studies either in *Medicago truncatula *mutants or via RNA interference approaches.

## Methods

MediPlEx extends the Sequence Analysis and Management System (SAMS), developed at Bielefeld University and combines it with the microarray expression datasets stored in TRUNCATULIX. An expression value for EST analyses is calculated my means of the logarithmic likelihood ratio introduced by Stekel *et al. *[17]. The software consists of four parts which are described in the following.

### SAMS

SAMS stores the TC sequences (assembled ESTs), the EST composition and sequence data of all TCs of the *Medicago truncatula *GeneIndex 10.0, generated at the J. Craig Venter Institute and annotations of all TCs created by an automatic annotation pipeline. The pipeline consists of several bioinformatics tools for gene annotation (BLAST against different sequence databases, Interpro, and HMMER) [44-46]. Using customized BLAST tools, the TCs were mapped to the reporters of the Mt16kOliPlus oligo-microarray chip, as well as to the Affymetrix Medicago GeneChip^® ^reporters.

### Logarithmic likelihood ratio

The logarithmic likelihood ratio developed by Stekel *et al. *[17] provides a statistical expression value for each TC for a unique combination of EST libraries. The logarithmic likelihood ratio is calculated as a ratio of ESTs assembled to a TC taking into account the total number of ESTs, the expression ratio of ESTs in each library and the size of the libraries. To calculate this R-value, the available libraries have to be divided into three groups: 'MUST contain ESTs', 'MUST NOT contain ESTs', and 'MAY contain ESTs'. The libraries and ESTs used for the calculation of the R-value are the ones marked as 'MUST contain ESTs' and 'MAY contain ESTs'. All TCs are then scanned for their composition of ESTs from these libraries. According to the ESTs and libraries, an R-value representing the expression is calculated for each TC.

For a more detailed description of the logarithmic likelihood ratio the reader is referred Stekel *et al. *.

### TRUNCATULIX

The TRUNCATULIX data warehouse serves as a data source for the microarray expression data used by MediPlEx. It was created in 2008 to allow fast and effective expression search in freely available *Medicago truncatula *sequence and expression data. The data warehouse covers over 100.000 sequences, combined with annotation data and BLAST results. Additionally, the results of over 200 microarray hybridizations are stored in the warehouse. These have been linked to the sequences using a BLAST homology search of the reporter sequences against the gene sequences.

### MediPlEx

MediPlEx integrates the results of different gene expression analysis methods to analyze them integratively to find new candidate genes and expression profiles. The user therefore first selects the EST libraries that should be used to filter the sequences. It is possible to select one of three states for each library: 'MUST contain ESTs', 'MUST NOT contain ESTs', and 'MAY contain ESTs'. 'MUST contain ESTs' means that the resulting TCs have to consist of at least one EST from these libraries. 'MUST NOT contain ESTs' denotes that the resulting TCs are not allowed to have an EST assembled from these libraries at all, and 'MAY contain ESTs' indicates that it does not matter if an EST is assembled from these libraries to a TC or not (see Figure 1). Different preselections for the libraries are available, some adopted from the DFCI website, while other are self-created. According to this selection, the TCs are scanned for their composition of ESTs from the libraries. For the TCs that match the query, the logarithmic likelihood is calculated (c.f. previous Section), to compute an expression value for the specific search.

In a second step (see Figure 2), the user selects the microarray experiments he wants to use for an expression analysis. For each of the TCs the results of the BLAST homology search against the two different microarray types Mt16kOliPlus and Affymetrix Medicago GeneChip^® ^are fetched from the SAMS database. These reporters are used to collect the expression datasets of the selected experiments from TRUNCATULIX. The resulting expression values (Mt16kOliPlus arrays: mean of the significance test, Medicago GeneChips^®^: a1mean) are listed in a table (Figure 3). All fetched expression values, as well as the calculated logarithmic likelihood ratio are used for a hierarchical clustering performed using the statistical analysis software R [47]. The result of the clustering is presented as a dendrogram (Figure 4). The user can then select to create two to eight cluster according to his estimation and the cluster dendrogram. The clustered genes are subsequently visualized in an interactive 3D application (Figure 5). Therefore, the user has to select three of the expression datasets, one for each of the three axis of the coordinate system. The genes are depicted as spheres in the coordinate system, different colors represent the associated cluster. Each cluster can be activated and deactivated for hiding the corresponding genes in order to gain a better overview. The coordinate system is rotatable and zoomable, the genes can be clicked to show the expression values of the experiments. A snapshot of the 3D view can be stored locally by clicking the "Save as png file"-button. The expression information and the clustering results can be exported as csv files, containing the annotation details of the TCs. A link provides direct access to the gene sequence and annotations stored in SAMS.

## Availability and requirements

Project name: MediPlEx

Project home page: http://www.cebitec.uni-bielefeld.de/mediplex

Operating system(s): Platform independent

Programming language: Perl, R

## Competing interests

The authors declare that they have no competing interests.

## Authors' contributions

KH initiated the project, implemented the backend and the frontend, computed the annotations for the sequence data, and is the main author of the manuscript. HK coordinated most of the Mt16kOliPlus microarray experiments and helped with the biological interpretation of the results. LJS implemented the 3D viewer. AG supervised the project. All authors revised and approved the final manuscript.

## Supplementary Material

Additional file 1 **File containing the 751 genes and expression values**. The .tsv file contains a table, separated using tab-stop as delimiter. The table stores the results of the expression analysis: The name of the found genes, the matching reporters of the two different layouts, the logarithmic likelihood ratio, as well as the expression values of the microarray expression experiments and the annotation of the genes. Click here for file

Additional file 2 **File containing the results of the clustering of the found genes**. The .tsv file contains a table, separated using tab-stop as delimiter. The table stores the four clusters created. Each cluster contains the gene names and the annotation of the genes. Click here for file

Additional file 3 **File containing the GO categories of the 4 created clusters**. The .xls file stores the GO categories of the genes in the four created clusters. Click here for file
